# Centrifugal Microfluidic Lateral Flow Assay Enables High Sensitivity Interleukin-6 Detection and Ultrafast Readout of Elevated Analyte Levels

**DOI:** 10.1021/acs.analchem.5c00413

**Published:** 2025-04-15

**Authors:** Daniel M. Kainz, Bastian J. Breiner, Anna Klebes, Nadine Borst, Roland Zengerle, Felix von Stetten, Tobias Hutzenlaub, Nils Paust, Susanna M. Früh

**Affiliations:** 1 Hahn-Schickard, Georges-Koehler-Allee 103, Freiburg 79110, Germany; 2 Laboratory for MEMS Applications, IMTEK - Department of Microsystems Engineering, University of Freiburg, Georges-Koehler-Allee 103, Freiburg 79110, Germany

## Abstract

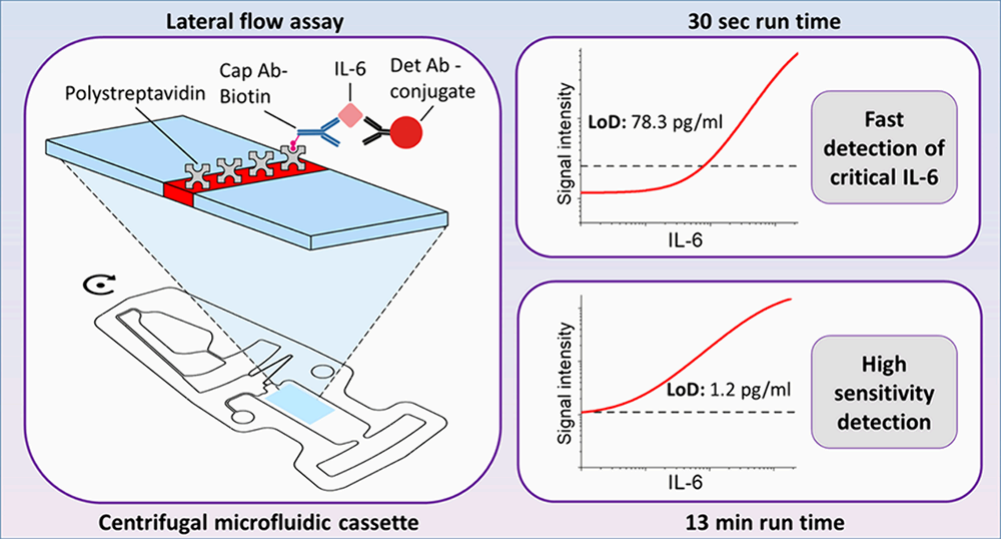

Balancing sensitivity and time to result is always a challenging part of the development of analytical tools and is of particular importance in clinical and point-of-care diagnostics. In this study, a highly sensitive fluorescence interleukin-6 lateral flow assay (LFA) was developed using centrifugal microfluidics. The sample flow rate through the lateral flow membrane is determined by centrifugal force, which can be precisely controlled with a processing device. Using this precise flow control, an ultrafast early readout after 30 s with a sensitivity of 78.3 pg/mL and a quantitative measurement up to 2000 pg/mL was achieved. Afterward, the flow rate was reduced, and thus, the incubation time increased to achieve a maximum sensitivity of 1.2 pg/mL within 13 min of run time. This high-performance LFA is intended to help particularly vulnerable patient groups, such as pregnant women and neonates, where a rapid and highly sensitive diagnosis of inflammatory biomarkers can make a life-saving difference. In addition to medical applications, the presented system can also be used for the analysis of binding kinetics directly on the lateral flow strip. This enables the development of lateral flow assays with the highest possible sensitivity in the shortest time. Therefore, this advancement leads to a new era of point-of-care testing with future prospects for fully automated centrifugal cassettes with enhanced performance.

Lateral flow assays (LFAs) have been in widespread use for decades and owe their popularity, among other things, to their ease of use and their relatively short time to result at the point of need. However, there are still severe limitations, especially in sensitivity and quantifiability in comparison to central laboratory tests.^1^ Many LFAs are only for qualitative or semiquantitative analysis, which is not sufficient for a variety of biomarkers that require quantitative analysis to unlock their full diagnostic potential, as is the case, for example, for inflammatory biomarkers.^2^

In particular, the detection of the pro-inflammatory cytokine interleukin 6 (IL-6) is useful in a wide variety of diseases.^3^ A recent meta-analysis determined the 95% confidence interval of IL-6 in healthy individuals as 4.631–5.740 pg/mL.^4^ However, it can rise by a factor of 10–100 during inflammatory processes and even up to >100,000 pg/mL in cases of septic shock.^5^ High concentrations need to be detected rapidly in order to properly treat the patient early on. In diseases like neonatal sepsis,^6^ chorioamnionitis,^7^ or pneumonia in patients with a cranio-cerebral injury,^8^ extreme inflammatory events take place, which can lead to severe complications if detected too late. This applies especially to vulnerable patient groups like neonates or unborn children.^9^ The low concentration in healthy individuals and the need for fast detection at elevated concentrations require a test with high sensitivity and ultrafast detection.

Currently, chemiluminescence^10^ and electrochemiluminescence immunoassays are used,^11^ which can achieve the sensitivity but require large instruments in the central laboratory. Enzyme-linked immunosorbent assays (ELISAs) can be used alternatively but require many manual steps and have a long detection time.^12^

As the demands on medical personnel become even more extensive, it is very important to make the handling and procedure of tests as simple and fast as possible. The necessity for manual steps or packaging and transportation of samples to the central laboratory can cause crucial delays.^13^ Therefore, a point-of-care (PoC) test with a very short time to result allows the medical personnel to stay at the bedside during the test and immediately access the diagnostic result afterward for efficient clinical decision-making.^14^ Quidel’s Sofia® 2 fluorescent immunoassay analyzer can reduce the run time of some classic LFAs to 3 min using its Advance Result Technology.^15^ Although this is a short time to result, the available LFAs are only qualitative, which is not sufficient for biomarkers like IL-6.

To our knowledge, the IL-6 LFA of Milenia Biotec is currently the only IL-6 PoC test available on the market (€17.90/test in January 2025). It has a dynamic range of 50–10,000 pg/mL IL-6 and is thus not able to detect slightly elevated IL-6 < 50 pg/mL, which can be important, e.g., in the detection of early onset neonatal sepsis.^6,16^ The signal generation is based on colloidal gold nanoparticles (Au-NPs) as antibody (Ab) labels, and the test can achieve quantitative results with the help of an LFA reader benchtop device in 20 min.^17^

Au-NPs are often used in LFAs because they provide an easy readout by eye and can be quantitative using a portable reader, but they do not enable the highest sensitivities. New innovative labels are also being explored for IL-6 LFAs to improve sensitivity. For instance, Huang et al. used europium nanoparticles (Eu-NPs) and achieved a sensitivity of 0.37 pg/mL IL-6 in a quantitative LFA in 15 min and only required a fluorescent LFA reader. It is noteworthy that in their LFA, the linear range only reaches up to 500 pg/mL, which can be problematic because the hook effect can lead to false low results in critical patients with very high IL-6 concentrations.^18^

In addition to using innovative labels, the sample volume can be increased to improve sensitivity. However, the classical LFA format is limited by the volume of the sample that can flow through the membrane. Therefore, Lei et al. used a vertical flow assay to increase the sample volume to 500 μL of 10-fold diluted serum and achieved a limit of detection (LoD) of 3.2 pg/mL IL-6 in 15 min.^19^ The system requires 11 manual steps, but the readout is semiquantitative without additional instruments, which keeps the initial costs low.

LFA sensitivity can also be increased by structural changes of the standard LFA design to reduce the overall flow rate passively by adding flow obstacles.^20,21^ The flow rate can also be actively decreased by centrifugal force. Hwang et al. showed that the wicking speed can be reduced using centrifugal force as a counter force,^22^ and a high sensitivity Troponin T assay was developed using this concept.^23,24^ By reducing the flow rate, the incubation time of the analyte at the test line is increased and, thus, those approaches achieve higher sensitivities, but consequently, the sample-to-answer time is increased. Therefore, there is still an unmet need for a PoC test that combines a high sensitivity with a fast sample-to-answer time, in particular, for higher concentrations that require immediate therapeutic actions.

One way to bridge the gap between assay sensitivity and short run times is the use of a flow control for LFAs by centrifugal microfluidics.^25^ We have published this concept in the past, and it is the technological basis of this publication. This technology necessitates a centrifugal device, which increases complexity and costs but also greatly increases the ease of use at the point of need, and it is designed for clinical environments where rapid and accurate diagnostics are prioritized. The LFA is performed under rotation, and the flow rate through the strip is precisely controlled by the rotational frequency. Compared to other integration strategies,^22,24^ this approach can not only decelerate but also accelerate the flow rate. This allows the incubation time of the assay to be increased to achieve higher sensitivities but also to be decreased to achieve rapid results using the same microfluidic cassette. Since the flow is not capillary-driven, it is independent of the substrate used. The microfluidic automation of the sample preparation steps,^26,27^ such as serial dilutions,^28^ blood plasma separation,^29,30^ or component prestorage^31,32^ were shown before. In a future development stage, these steps can be combined with our integration strategy to achieve a fully automated sample-to-answer PoC test, which entails pipetting a blood sample into the cassette, inserting the cassette into the device, and reading the results from the device.

In this work, we present a lateral flow assay integrated into a centrifugal microfluidic cartridge, enabling rapid and highly sensitive IL-6 detection. Unlike our previous studies, which focused on the centrifugal microfluidic working principle, we optimized the assay performance by scaling the flow rate during the run. A high initial flow rate enables an ultrafast early readout, while subsequent deceleration extends the incubation time, allowing detection across the clinically relevant IL-6 range. Using fluorescence beads and a polystreptavidin test line, we achieve both a rapid response and high sensitivity, demonstrating for the first time an IL-6 assay that combines these critical features.

## Experimental Section

### Assembly of the Lateral Flow Membrane Strips

**Figure 1:**
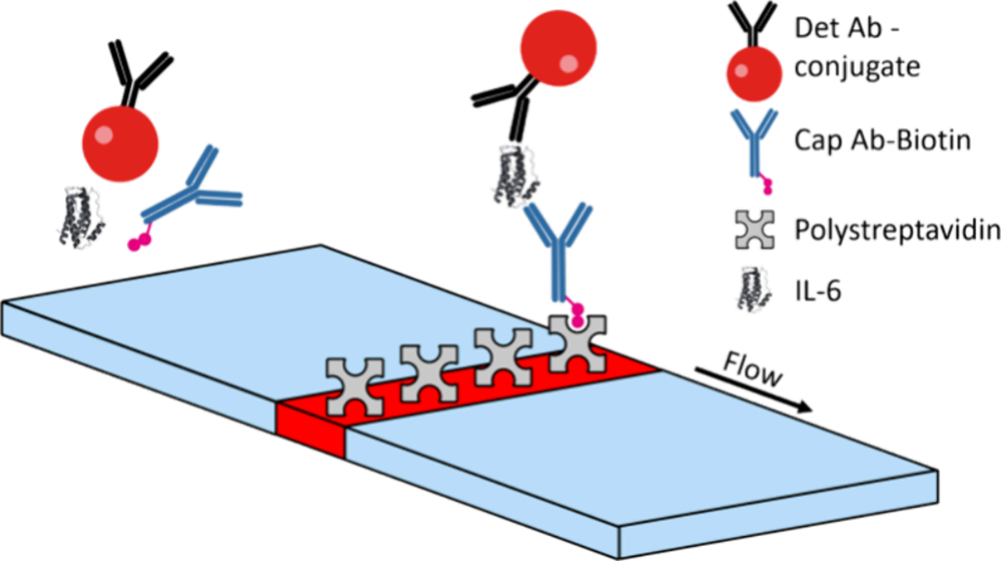
Sandwich-type immunoassay design with detection antibody—FluoSphere conjugate (Det Ab-conjugate) and biotinylated capture antibody (Cap Ab-biotin) in the assay mix and polystreptavidin on the test line.

The assay design of the sandwich type LFA for human IL-6 is shown in Figure 1. The backed CN140 nitrocellulose membrane (Sartorius AG, Germany) was cut into 92 mm long sheets, and double-sided adhesive film 8153 300 LSE (3M, USA) was added to the backing. The test line was added by dispensing 200 μg/mL Polystreptavidin R (BioTez, Germany) in PBS using an AD3220 line dispensing system (BioDot, USA) at a rate of 1 μL/cm. The sheets were dried for 1 h at 37 °C and stored at room temperature (RT) with desiccant until further use. The sheets were acclimatized to a relative humidity of >50% and cut to a length of 8 mm. A 20 kN HKP/L DS manual toggle press with air assistance (Gechter, Germany) with knives at a 4.4 mm interval was used to stamp the sheets to the depth of the secondary liner. The 4.4 mm wide and 8 mm long membrane strips were peeled off and attached to the microfluidic cassette.

### Antibody Functionalization

The two monoclonal mouse antihuman-IL6 antibodies (Abs) were provided by Milenia Biotec (Gießen, Germany). The capture Ab 10C3 was biotinylated at a concentration of 1.5 mg/mL using the EZLink Sulfo-NHS-LC-Biotin (Thermo Fisher Scientific, Germany) at a 23.3-fold molar excess of biotin reagent to Ab for 30 min at RT in PBS. The biotinylated Ab was washed with PBS two times with a VivaSpin 500 30 kDa molecular weight cut-off (Sartorius, Germany) and once with 0.09 % NaN_3_ in PBS, and the biotinylation ratio was measured via the fluorescence biotin quantitation kit (Thermo Fisher Scientific, Germany) (see the Supporting Information). The detection Ab 8A11 was conjugated to FluoSpheres^TM^ carboxylate-modified microspheres, 0.2 μm, red fluorescence (580/605) (Life Technologies GmbH, Germany) with the use of 1-ethyl-3-(3-(dimethylamino)propyl)carbodiimid (EDC) and *N*-hydroxysuccinimid (NHS) (Sigma-Aldrich, USA). 5 μg of the Ab and 100 μg of the beads were mixed in 390 μL of 50 mM 2-(*N*-morpholino)ethanesulfonic acid (MES) buffer pH 5.5. 5 μL of 2.1 mM EDC and 5 μL of 3.5 mM NHS were added; the reaction mix was incubated for 2 h on a rotary mixer at RT. 75 μg of ethanolamine were added to quench the reaction, and the mix was incubated for 30 min. The conjugate was centrifuged for 9 min at 16,000 rcf and washed with 400 μL of storage buffer (PBS containing 0.05% (v/v) Tween 20, 0.1% (w/v) bovine serum albumin heat shock fraction (hs-BSA), 0.03% (w/v) Synperonic P84, and 0.05% (w/v) sodium azide). The conjugate was centrifuged again and stored in storage buffer at 4 °C.

### Fabrication and Design of the Centrifugal Cassette

**Figure 2:**
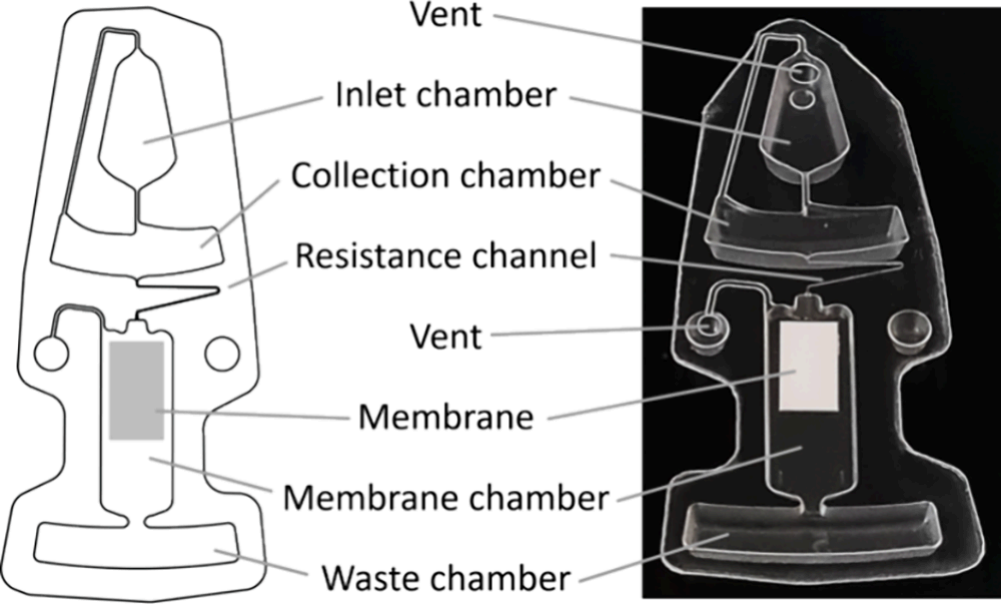
Schematic sketch and photograph of the microfluidic cassette with an integrated membrane.

A centrifugal cassette has been developed with an integrated lateral flow strip, as shown in Figure 2. In order to avoid bypass flows around the strip, the cassette follows the previously published design guideline for the integration of LFAs in a centrifugal force field.^25^ The cassettes were designed in SolidWorks (Dassault Systèmes, France).

The inlet chamber holds the 50 μL of assay mix. The collection chamber serves as a sample reservoir during the rotation. The lateral flow membrane strip is attached to the bottom of the membrane chamber without contact to the side walls or the sealing foil. The radially outermost chamber is the waste chamber. The resistance channel between the collection chamber and the membrane chamber is 74 μm wide and 67 μm high and has a length of 15.3 mm. The corresponding design factor *D* is 0.88.^25^ All chambers were vented.

The centrifugal cassettes were microthermoformed^33^ by the Hahn–Schickard Prototyping for microfluidic chips using a 188 μm-thick ZF 14 cycloolefin polymer film (Zeon Chemicals, USA). The lateral flow membrane strips were adhered to the membrane chambers before the cassettes were sealed with pressure-sensitive adhesive foil 9795 R (3 M, USA). The sealed cassettes were then CO_2_-laser cut with a VLS4.75 (Universal laser systems, USA) to a final shape and stored at RT with desiccant.

### Sample Preparation and Processing

The centrifugal cassettes were positioned with two alignment structures and attached to a rotor of a custom centrifuge (BioFluidix, Germany) with adhesive tape (Supporting Information). Fifteen cassettes were processed in one run. The final inner radial position of the membrane strips in the rotor was 102 mm.

**Figure 3:**
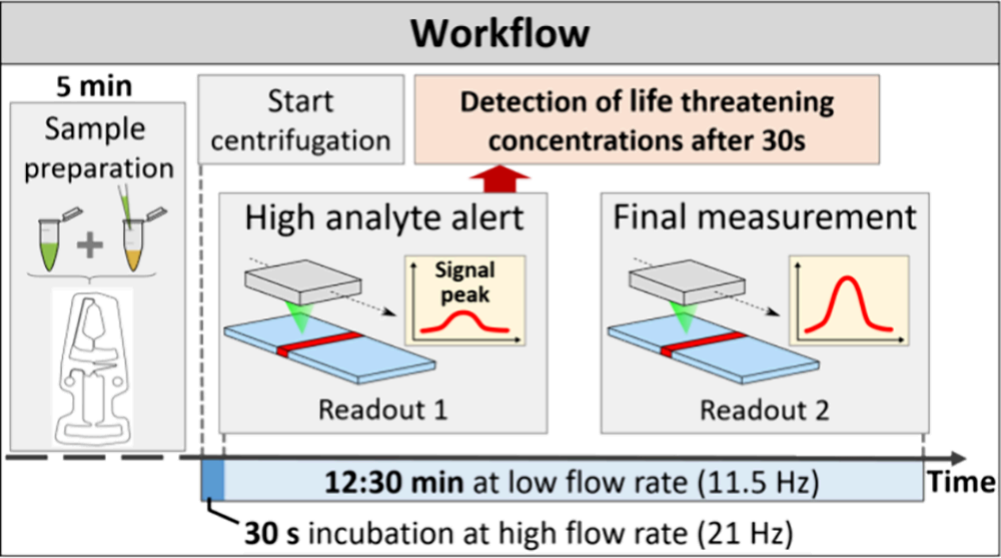
Workflow of the IL-6 centrifugal assay with readout 1 (after 30 s) and readout 2 (after 13 min).

Recombinant human IL-6 protein (R&D Systems, USA) was spiked into human serum (HyTest, Finland) at different concentrations (2–2000 pg/mL). 0.0067% (w/v) FluoSphere-8A11 conjugate (refers to the microsphere concentration) and 0.27 μg/mL biotinylated 10C3 capture Ab were diluted in buffer (PBS containing 3.33% (v/v) Tween 20, 10% (w/v) hs-BSA, 2.5% (w/v) Pullulan (Polysciences, USA)) to obtain the concentrated conjugate solution. For each test, 15 μL of the conjugate solution (including capture Ab and FluoSphere conjugate) were mixed with 35 μL of the spiked serum samples. The resulting 50 μL of assay mix were pipetted into the inlet chamber of the centrifugal cassette. The time between mixing the sample with the Abs and the start of the centrifuge run was set to 5 min to allow for the manual handling steps of 15 tests. The rotational frequency was increased to 15 Hz at 5 Hz/s for 4 s to prime the structure. The frequency was then accelerated to 21 Hz at 5 Hz/s to achieve a high flow rate. After a rotation time of 30 s, a readout of all 15 strips was performed at a standstill. The readout of each strip took about 3 s. The rotational frequency was then increased to 11.5 Hz at 5 Hz/s to achieve a total run time of 13 min. After that, a second readout was performed at a standstill (Figure 3). Additionally, a second run in which the frequency was kept at 21 Hz for a total run time of 5 min was performed. Measurements were recorded after 30 s and at the end of the 5 min run. All runs and readouts were performed at RT.

### Readout and Analysis of the Strips

The readout was performed automatically by a confocal fluorescence detector FSENS90 LF (Dialunox, Germany) attached to the centrifuge. The detector, which is commonly used in a commercial PoC lateral flow reader, was moved 6 mm in the radial direction at a speed of 4 mm/s across the test line. To obtain the signal intensity of the test line, the measured peak intensity values were analyzed with an automated Python 3.6 script using the PeakUtils 1.3.3 package. An image of the test line was taken with a fluorescence microscope Observer Z1 (Zeiss, Germany) for quality control.

The signal coefficient of variance (CV) was calculated by dividing the standard deviation by the mean value of the measured signal intensity. The total signal CV includes all measurements above the LoD. The LoD was calculated as the intersection between the mean signal intensity of the blank plus three times its standard deviation and the assay fitting curve. Due to the sigmoidal shape of the assay a logistic fit function was chosen as shown in eq 1:
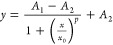
1where *A*_1_ is the initial value, *A*_2_ is the final value, *x*_0_ is the center value, and *p* is the power of the logistic function. The logistic function was fitted to the measured data points by using default parameters using the Levenberg–Marquardt solver in Origin (OriginLab Corporation).

### Flow Rate Measurement

The mean flow rate through the membrane was calculated by dividing the sample volume of 50 μL by the total flow time. The total flow time is the time interval between reaching the processing frequency and emptying of the inlet chamber. The start and end times were identified using a stroboscopic setup in the custom centrifuge. Five samples were processed at 21 and 11.5 Hz to measure the flow rate.

### Viscosity Measurement

The viscosity of the assay mix was measured at 24 °C by using an MCR101 rheometer (Anton Paar GmbH, Germany).

## Results and Discussion

### Centrifugal Cassette and Flow Rates

All 57 manufactured cassettes were functional and were processed. In contrast to other LFA-integration strategies,^22−24^ the flow through the strip is completely controlled by centrifugal force. As a result, the flow rate can be increased or decreased by the rotational frequency (see the Supporting Information for a video of fluidic processing).

As illustrated in Figure 3, the assay starts at a frequency of 21 Hz with a high mean flow rate of 10.77 ± 0.23 μL/min (μ ± SD). This high flow rate is particularly notable given the high viscosity of the assay mixture of 1.83 ± 0.09 mPas (μ ± SD) at 24 °C. In a standard capillary force-driven LFA, this high viscosity reduces the flow rate by a factor of ∼10, resulting in an undesirably long run time (1.07 ± 0.03 μL/min; 47 min run time for 50 μL; see the Supporting Information). After 30 s, the centrifuge is stopped, resulting in no flow through the strip. At this point, an automated readout was performed. The aim of this readout after 30 s of run time is to detect high concentrations of IL-6, which can quickly inform the user if a patient requires immediate attention.

**Figure 4:**
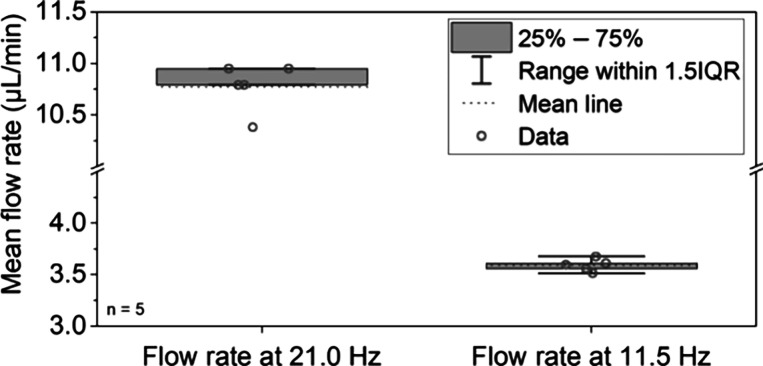
Measured mean flow rates through the cassette of a 50 μL sample at 21 and 11.5 Hz. Each gray circle represents a measured value.

After the first readout, the frequency was reduced to 11.5 Hz to decrease the mean flow rate to 3.59 ± 0.06 μL/min (μ ± SD). By doing so, the flow rate of the remaining sample was reduced by a factor of 3 to increase the incubation time and thus the sensitivity of the assay. The flow rate measurements are plotted in Figure 4.

**Figure 5:**
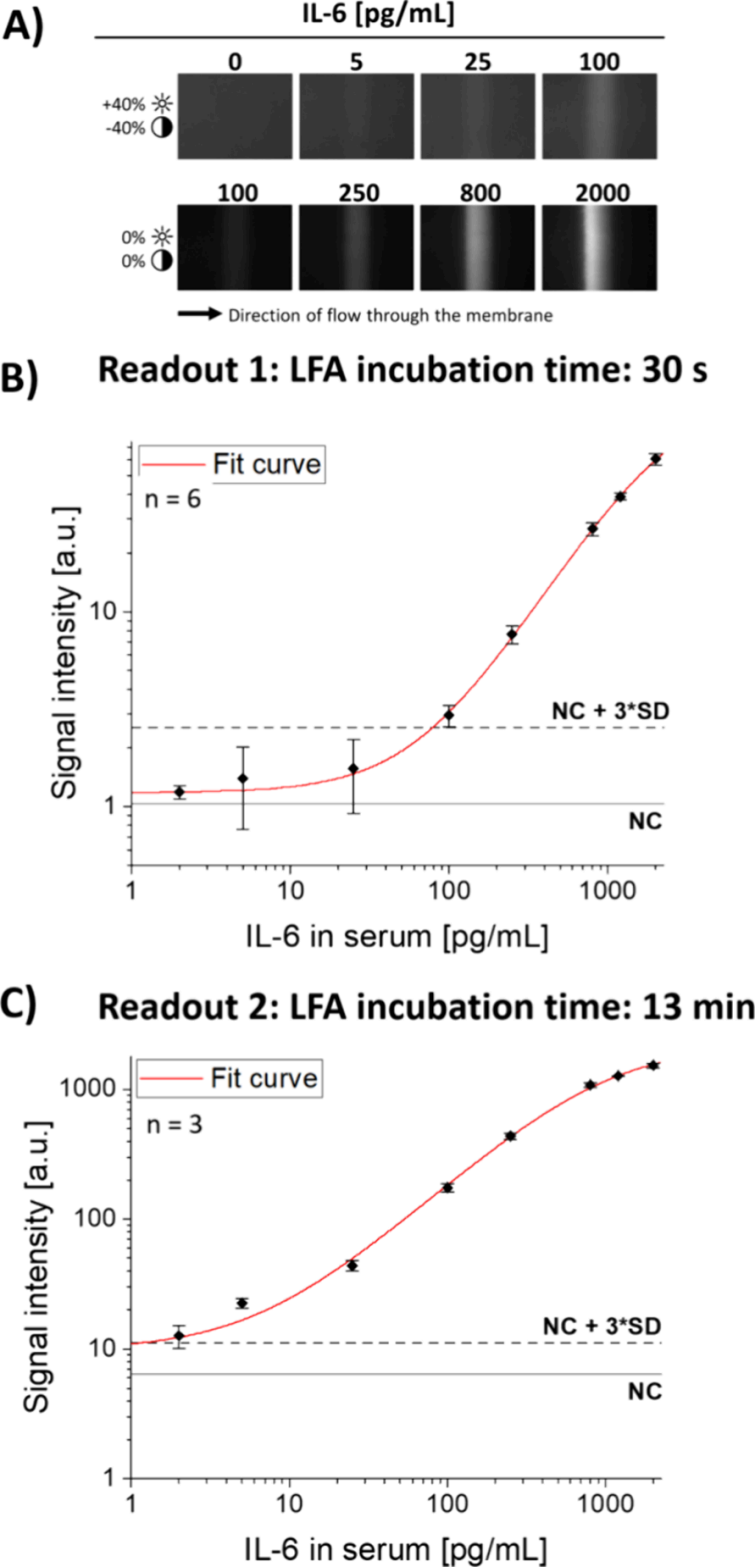
Overview of assay results. (A) Microscopic fluorescence images of the test lines after 13 min for quality control. Contrast and brightness adjustments were applied to enhance the visualization of low-intensity signals inherent in the 12-bit original image, ensuring their clarity for interpretation without altering the data integrity. (B) Plot of the measured fluorescence signal intensities after 30 s (readout 1). (C) Plot of the measured fluorescence signal intensities after 13 min incubation time (readout 2).

No bypass flow around or across all membranes was observed (100% success rate), which would cause irregularities in the test line as was shown in previous work.^25^ The resulting test line homogeneity across the width, as shown in Figure 5A, indicates an evenly distributed flow across the membrane width. Thus, the described centrifugal system allows for precise control of the incubation time of the IL-6 immunoassay.

During the development of a standard capillary force-driven LFA, a membrane is selected with the best combination of binding and flow properties for the assay. The internal surface area of membranes is inversely proportional to their pore size. This, in turn, results in a higher protein binding capacity for membranes with smaller pore sizes.^34^ Increasing the amount of captured Ab on the test line can be used to enhance the sensitivity and dynamic range of the assay. However, reducing the size of membrane pores, and therefore increasing protein binding capacity, always leads to higher fluidic resistances and thus longer assay times. Compared to the classical LFA, the centrifugal flow controlled LFA allows short assay times despite small pore sizes because the flow is driven by the centrifugal force, which can be adjusted by the rotational speed. Therefore, LFA designers are freed from selecting membranes based on their flow properties and can choose membranes solely on the basis of their binding capacity to enhance the assay performance without increasing assay run time.

The centrifugal cassette effectively demonstrates the ability to adjust flow rates through the LFA membrane, thereby fine-tuning assay incubation times, and provides LFA developers with valuable tools to overcome the current limitations of classical LFA design.

### Readout of Elevated IL-6 Levels after 30 s

During the first 30 s, the sample liquid flows through the strip at a high flow rate to allow for rapid detection of samples with particularly high IL-6 concentrations. Since the collection chamber is filled completely at the beginning, the centrifugal pressure is higher at the beginning than in the end. Therefore, the flow rate within the first 30 s exceeds the average of 10.77 μL/min. This rate is not directly measurable during operation, so it was estimated by simulation^35^ to be 17.2 μL/min, resulting in 8.6 μL of assay mixture passing through the strip during the first 30 s.

The LoD for the early readout 1 was calculated to be 78.3 pg/mL IL-6, and the assay was able to measure the samples ranging from 100 to 2000 pg/mL, as shown in Figure 5B. The average signal CV of all measured concentrations above the LoD was 8.5%. The data follow a linear progression up to 2000 pg/mL. With this performance, the initial measurement can be used to produce a rapid quantitative high analyte alert because it can detect strongly elevated IL-6 concentrations in critical patients. The sensitivity of the early readout measurement is also already sufficient for specific diseases, e.g., for the prediction of early onset neonatal sepsis, a cut off of 108.5 pg/mL IL-6 in umbilical cord blood was proposed.^36^

The ability of the assay to detect a concentration of 78.3 pg/mL after a run time of 30 s using only 8.6 μL of the assay mix is attributed to two main factors. First, the slower formation of the partial immunoassay complexes compared to the streptavidin–biotin binding reaction can already take place during the 5 min of sample preparation. The sample preparation time includes mixing the serum sample with the Ab conjugates, pipetting the sample into the cassettes, and inserting the rotor into the centrifuge. Second, the high initial flow rate allows only a short contact time of the immunocomplexes with the test line. Therefore, a biotin–streptavidin binding reaction was chosen for the test line because the affinity of streptavidin for biotin^37^ is higher than the affinity of antibodies for their antigens.

When comparing the speed of different diagnostic tools, it is important to consider the total time to result, including sample preparation and preincubation steps. In a future development for a convenient and user-friendly detection of IL-6, a blood plasma separation, all metering and mixing steps, and the resuspension of the prestored Ab conjugates could be automated in the microfluidic cassette. Since the flow rate through the strip can be increased, thereby reducing the run time of the LFA, this technology has the potential to reduce the overall time to result in a fully automated system.

The Sofia® 2 fluorescent immunoassay analyzer (Quidel, USA) requires several manual sample preparation steps and performs its first readout after a flow time of 3 min for some of their LFA products.^15^ Compared to this, we could reduce the minimum flow time by a factor of 6 to 30 s. Therefore, we see the potential in the centrifugal integration strategy to not only perform a readout as the Sofia® 2 analyzer but also automate the entire sample-to-answer workflow as described above within 3 to 5 min.

Using centrifugal microfluidic flow control, we could reduce the minimum flow time to measure life-threatening IL-6 concentrations above 78.3 pg/mL to 30 s.

### Final High-Sensitivity Readout after 13 min

After the initial 30 s of run time and the first readout, rotation of the cassettes was resumed. To achieve a higher sensitivity with the remaining sample, the rotational frequency was set to 11.5 Hz to increase the total run time on the test strip to 13 min. Using the data from the second readout, the LoD was calculated to be 1.2 pg/mL, a 65-fold improvement compared to the readout after 30 s. The mean signal CV was 9.4%. No discrepancy in results was observed compared to those obtained after 30 s. The assay curve is plotted in Figure 5C. Fluorescence microscopy pictures of exemplary test lines after 13 min are depicted in Figure 5A for visual comparison.

The signal on the test line becomes saturated at higher IL-6 concentrations with extended run time. A linear progression of the signal intensity up to 800 pg/mL IL-6 can be observed, which starts to saturate at analyte concentrations of >800 pg/mL IL-6. Since the data from readout 1 after 30 s do not exhibit a saturation tendency up to 2000 pg/mL IL-6, it can be concluded that even a higher upper limit of quantification can be achieved. By combining the measurements after 30 s and after 13 min, a total dynamic range of 2–2000 pg/mL is attainable. Currently, the presented test cannot cover the upper physiological range of IL-6. A further development could incorporate an automated dilution and execution of a second LFA on the same cassette to also cover concentrations up to 200,000 pg/mL.

With the very high sensitivity and a sample volume of only 35 μL serum, our results far exceed the performance of the only commercially available LFA for IL-6.^18^ We additionally performed our centrifugal LFA with the Au-NP conjugate from the commercial LFA to highlight the importance of fluorescence signal transduction in obtaining the high sensitivity (see the Supporting Information). In the scientific literature, similar sensitivities were achieved employing novel labeling techniques, e.g., Huang et al. using Eu-NPS.^18^ Our advances in microfluidic actuation and assay integration improve transport dynamics and reaction kinetics rather than signal generation. Therefore, integrating this work with more advanced labeling to further increase the sensitivity is feasible. Huang et al. achieved an upper dynamic range of 500 pg/mL, which cannot cover all clinically relevant IL-6 concentrations and is susceptible to the hook effect.^18^ An increase in the upper dynamic range in the classical LFA format is difficult to realize without additional manual steps and without compromising sensitivity. This illustrates the significance of increasing our upper dynamic range from 800 to 2000 pg/mL by combining the 2 readouts and the potential for an automated dilution step in the next iteration.

The vertical flow assay by Lopez-Calle et al.^23^ showed similar performance but required 11 manual handling steps. Our test requires only one mixing step before injection into the cassette with no additional washing steps. As the automation of sample preparation and assay execution is a key advantage of centrifugal microfluidics, the outlook for our system is a fully integrated cassette that only requires sample addition before entering into the processing device. An overview of the mentioned IL-6 POC tests is shown in Table 1 for better comparison.

**Table 1:** Selection of PoC Assays for the Detection of IL-6

**methodology**	**sensitivity [pg/mL]**	**linear range [pg/mL]**	**run time [min]**	**sample**	**no. of manual steps**	**instrument requirements**
commercial LFA with Au-NPs^17^	50	50–10,000	20	100 μL serum, plasma or amniotic fluid	2–3^a^	optical LFA reader or evaluation card
LFA with Eu-NPs^18^	0.37	2–500	15	70 μL serum	2	fluorescence LFA reader
vertical lateral flow^19^	3.2	10–10,000	15	50 μL serum (1:10 diluted to 500 μL)	11	semiquantitative readout by eye
centrifugal LFA/30 s readout (this work)	78.3	100–2000	0.5	6 μL serum	3	programmable centrifuge with fluorescence sensor
centrifugal LFA/13 min readout (this work)	1.2	2–800	13	35 μL serum	3	programmable centrifuge with fluorescence sensor

a The performance with evaluation card takes one additional step.

With the help of centrifugal flow control, a LoD of 1.2 pg/mL could be achieved after a total run time of 13 min. The high sensitivity was achieved by using a sample volume of only 35 μL of serum. The developed PoC test is therefore not only able to compete with central laboratory analysis in terms of sensitivity but is also particularly suitable for neonatal diagnostics, where a reduction in sample volume is a decisive advantage. For the end user, only a simple benchtop device with a motor for rotation and a movable detector is required, making it both accessible and easy to use in various clinical settings.

### Run Time vs Sensitivity

In addition to its diagnostic applications, the system offers the ability to analyze binding reactions directly on the test strip. Such analysis can be used to achieve the highest possible sensitivity in the least amount of time. The centrifugal flow control facilitates this optimization by enabling the isolated analysis of the run time and assay performance correlation. This is possible because the flow rate and, thus, the incubation time can be adjusted independently of other influencing variables, such as pore size or the viscosity of the assay mix. In the following, we illustrate this capability by comparing the assay results with incubation times of 5 and 13 min.

The sensitivity of a LFA is dependent on many factors related to the three central physicochemical processes—convective flow, diffusion, and binding reactions in the test strip.^38,39^ Since analyte diffusion in nitrocellulose^40^ is much faster than the respective chemical reactions, it can be safely omitted from the following considerations of real-world LFAs.^41^ The reactants are transported to the test line by the convective flow, which means that the amount of bound immunocomplex on the test line at any point in the run is dependent on the balance between flow rate and reaction rate.^38^ The signal measured on the test line is proportional to the amount of bound immunocomplex consisting in our case of detection Ab, analyte, biotinylated capture Ab, and polystreptavidin. The reaction rate for the formation of the immunocomplex is based on the concentrations of the involved molecules and their respective association and dissociation constants.

If the reaction rate is low and the flow rate is high, the formation of the bound immunocomplex will be reaction-limited, which means that the incubation time of the reactants in the test line zone will not suffice to bring the binding reactions close to saturation.^39^ If the reaction rate is high and the flow rate is low, it will be in a transport-limited regime with a boundary layer in the test line zone where the concentrations of the reactants are decreased, and thus, the binding reaction will be decelerated.^39^ To achieve the highest possible sensitivity in the fastest possible time, the flow rate of an assay should be tuned so that the formation reaction of the immunocomplexes on the test line is just about saturated (dependent on analyte concentration).

After the initial 30 s, only a portion of the sample volume flowed through the strip at a high flow rate. Therefore, we showcase an additional experiment where the rotational frequency of the first 30 s was kept high until the complete sample flowed through the strip. This resulted in a total run time of 5 min. All test strips were processed successfully in this experiment. Over the concentration range from 25 to 2000 pg/mL, the mean signal intensity was increased by a factor of 3.0 with the longer incubation time of 13 min, while the mean signal intensity of the blank was only increased by a factor of 1.2.

**Figure 6:**
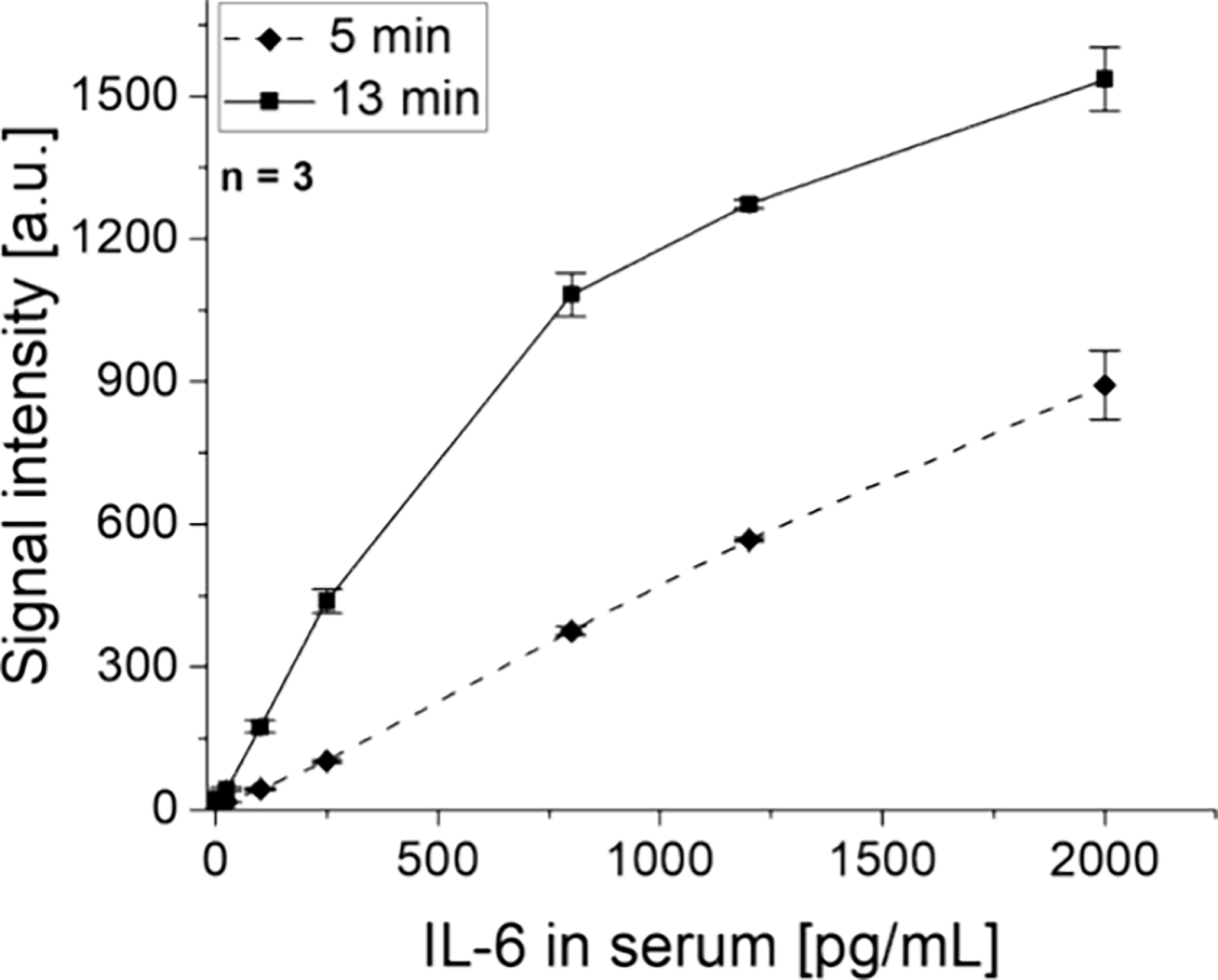
Plot of the measured fluorescence signal intensities of a 50 μL assay mix after a total run time of 5 and 13 min (data of the 13 min plot similar to Figure 5C).

The concentration curve of the assay after 5 min showed a linear progression up to 2000 pg/mL of IL-6, where a quantification of the assay result is possible in an optimal way (see Figure 6). In contrast, the slope of the concentration curve of the standard assay with a run time of 13 min starts to decrease after 800 pg/mL IL-6 in the linear representation of the data. This indicates that the linear dynamic working range of the immunoassay can be adjusted by changing the incubation time on the test line. With a shorter incubation time, the saturation of the signal happens at a higher analyte concentration, thus increasing the linear progression on the upper end of the calibration curve.

By reduction of the flow rate, the incubation time was increased and the sensitivity was enhanced. With an increased flow rate, the time to result is reduced, and the linear range is shifted to higher concentrations. Combining both flow rates in one test could lead the way in diagnostic systems, which are capable of adjusting their parameters to the properties of an individual sample to provide the best performance for each sample. The comparison of the data from the different total run times shows that an existing immunoassay can be fine-tuned only by changing the flow rate independent of test strip geometry or assay components.

## Conclusions

The limitations of conventional capillary-driven lateral flow strips were overcome by employing centrifugal flow control. This allowed the development of a high-performance IL-6 LFA with a very high sensitivity of 1.2 pg/mL and an ultrafast readout of elevated concentrations within 30 s. The assay requires a benchtop device, and it is designed for clinical settings where rapid and accurate diagnostics are essential. The fluorescence signal transduction and the interplay between biotin–streptavidin binding at the test line and controlled flow rate are all important factors to achieve analytical performance.

After only 30 s, IL-6 concentrations between 100 and 2000 pg/mL could be measured quantitatively using a high flow rate of 10.77 μL/min. The performance of the test after a run time of 30 s is suitable for a wide range of diagnostic questions, providing a real benefit in terms of speed and convenience for the end user.

If a sample contains less IL-6 than the early readout threshold, then the run continues until the total run time of 13 min is reached. The system can then achieve a sensitivity of 1.2 pg/mL IL-6 using a human serum sample of only 35 μL and a flow rate of 3.59 μL/min. Therefore, all relevant diagnostic questions can be answered by using the developed PoC test.

The centrifugal flow control can also be employed to study binding kinetics, which can be used to fine-tune the assay to achieve the highest possible sensitivity in the shortest possible time. In addition, the linear range of the assay can be adjusted independently of the test strip and reagents simply by changing the flow rate via the centrifugal frequency.

This work paves the way for a new generation of PoC tests, combining rapid results for emergency cases with the high sensitivity required for many analytes. The next step would be a fully automated sample-to-answer centrifugal cassette to test blood samples directly, creating a convenient, high-performance PoC test to be used by hospital clinicians.

## Supporting Information Available

Biotinylation results, custom centrifuge picture, and details on fit curve models, including Au-NP experiments (PDF)
Video of the fluidic processing (MP4)
