# Correction to “Centrifugal Microfluidic Lateral Flow Assay Enables High Sensitivity Interleukin‑6 Detection and Ultrafast Readout of Elevated Analyte Levels”

**DOI:** 10.1021/acs.analchem.5c02837

**Published:** 2025-06-30

**Authors:** Daniel M. Kainz, Bastian J. Breiner, Anna Klebes, Nadine Borst, Roland Zengerle, Felix von Stetten, Tobias Hutzenlaub, Nils Paust, Susanna M. Früh

There are two corrections to the original paper:

On page 8988, line 61 (right column), the reference number “18” should be replaced with the number “17”.

On page 8989, line 18 (left column), “Lopez-Calle et al.^23^” should be replaced with “Lei et al.^19^”

These corrections have no impact on the findings of the original manuscript. We regret any confusion caused by this error.

