# Supplementary material for: Centrifugal Microfluidic Lateral Flow Assay Enables High Sensitivity Interleukin-6 Detection and Ultrafast Readout of Elevated Analyte Levels
Source: Anal Chem. 2025 Apr 15;97(16):8984–91. doi: 10.1021/acs.analchem.5c00413 (PMC12044588; doi:10.1021/acs.analchem.5c00413)
Supplement: Supplementary file 1 — ac5c00413_si_001.pdf [file ac5c00413_si_001.pdf]

Supporting Information

Centrifugal microfluidic lateral flow assay enables high sensitivity interleukin-6 detection and ultrafast readout of elevated analyte levels

Daniel M. Kainz[1], Bastian J. Breiner[1], Anna Klebes[1,2], Nadine Borst[1,2], Roland Zengerle[1,2], Felix von Stetten[1,2], Tobias Hutzenlaub[1,2], Nils Paust[1,2*], Susanna M. Früh[1]

[1] Hahn-Schickard, Georges-Koehler-Allee 103, 79110 Freiburg, Germany

[2] Laboratory for MEMS Applications, IMTEK - Department of Microsystems Engineering, University of Freiburg, Georges-Koehler-Allee 103, 79110 Freiburg, Germany

Corresponding author: Phone: +49 761 203-73245

Fax: +49 761 203-73299

E-mail: nils.paust@imtek.uni-freiburg.de

Table of Content

Biotinylation results S2

Custom centrifuge S3

Run time measurement dipstick S4

Logistic fit curve models S5

Gold nanoparticle lateral flow assay S8

Biotinylation results

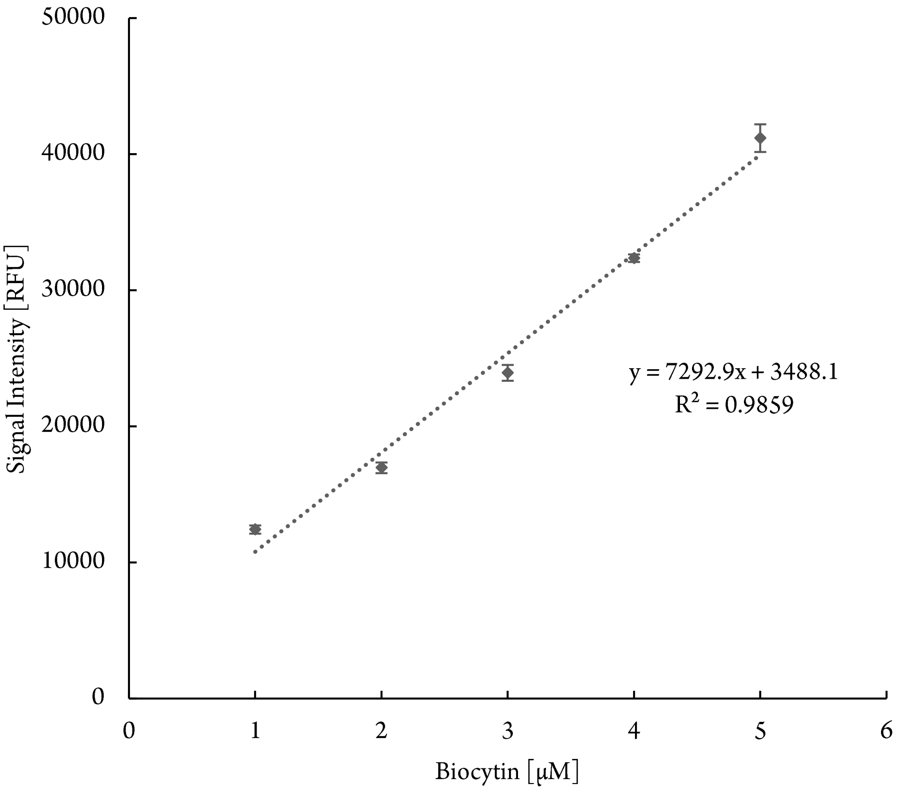

Figure S1. Biocytin standard curve created with Fluorescence Biotin Quantitation Kit (Thermo Fisher Scientific, Germany). Depicted are mean values with Standard deviations.

Table S1. Fluorescence Biotin Quantitation Kit results for the Biotin/Ab ratio of the 8A11 capture Ab after biotinylation with EZLink™ Sulfo-NHS-LC-Biotin (Thermo Fisher Scientific, Germany)

Sample	c(Ab) [µM]	Mean [RFU]	SD [RFU]	CV [%]	c(Biotin) [µM]	Biotin/Ab ratio
8A11 capture Ab	1.33	29295	539	1.84	(29295 – 3488.1) / 7292.9 = 3.54	3.54 µM / 1.33 µM = **2.65**

Custom centrifuge

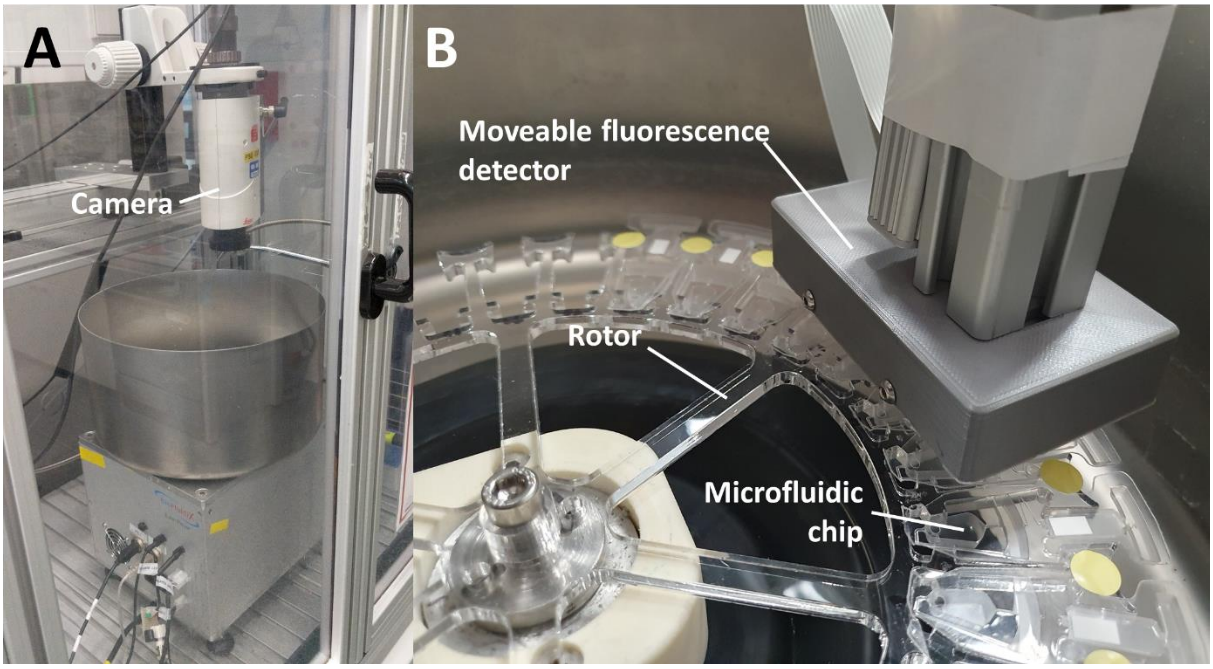

Figure S2. *A)* Centrifuge used in this project. The customized device was manufactured by BioFluidiX (Freiburg, Germany) as part of a previous project and weighs around 20kg with a size of 40 cm x 35cm x 45 cm (L x W x H). It is equipped with a camera and a stroboscopic light. *B)* View inside the centrifuge: all the microfluidic chips are attached to a rotor that can hold up to 30 chips. The fluorescence detector automatically moves over the lateral flow strips to detect the signal.

Run time measurement dipstick

The run time of the assay mix used for the centrifugal LFA was tested in the dipstick format to highlight the potential for an increase of flow rate when replacing capillary force with centrifugal force. Since the flow rate is constant in the centrifugal LFA but not in the dipstick format, it was decided to record the run time instead for better comparability. 50 µL of the assay mix (70 % human serum, 1 % Tween20, 3 % hs-BSA, 0.75 % Pullulan, 1 % red ink in PBS) was pipetted into a MTP and dipsticks consisting of nitrocellulose membrane CN140 (Sartorius, Germany), waste pad CF5 (Cytiva, USA) and backing card KN-V1090.1 (Kenosha, The Netherlands) were added. The liquid flow was videotaped and the time determined when the front of the liquid stopped moving (See Figure S3). The experiment was performed in triplicate and a **mean run time of 46 min and 50 s** with a standard deviation of 79 s was recorded.

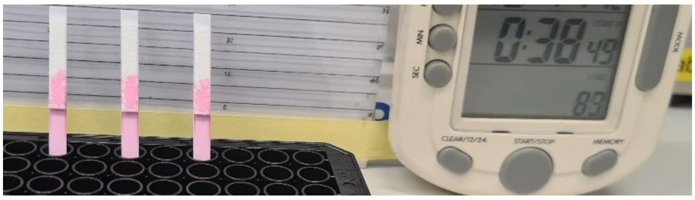

Figure S3. Experimental setup for recording the dipstick run time.

Logistic fit curve models

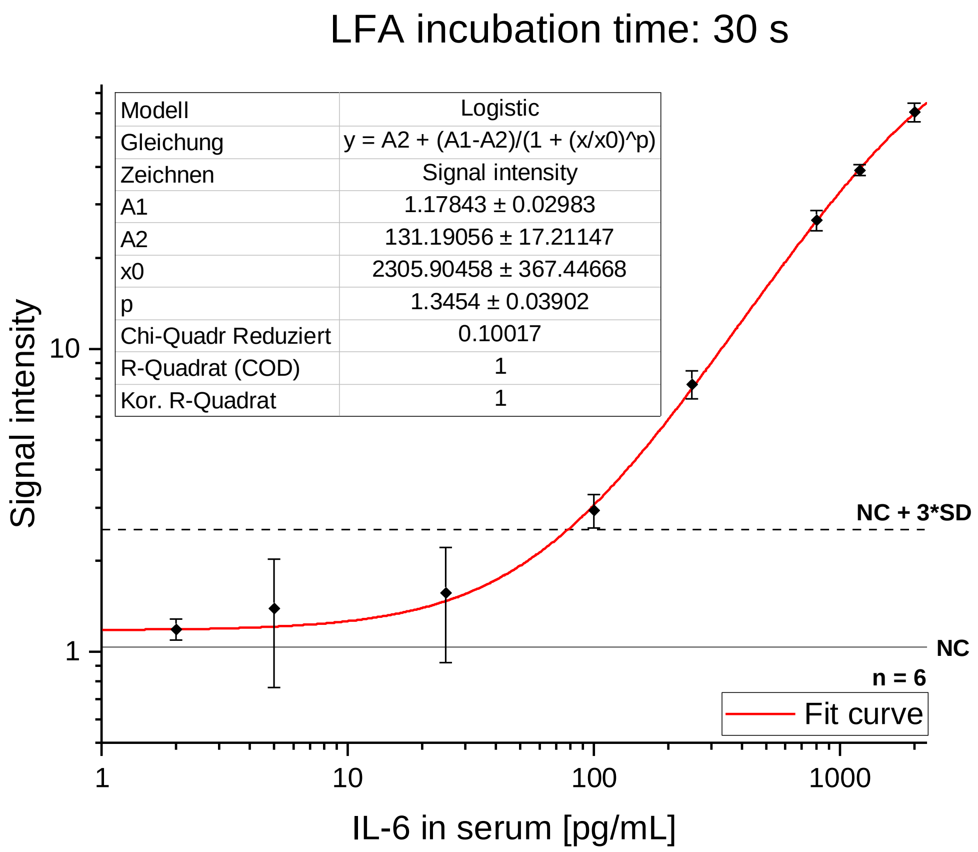

Figure S4. Measured points and fit curve of the centrifugal IL-6 assay after 30 s incubation time. The logistic fit curve model parameter are shown in the upper left corner.

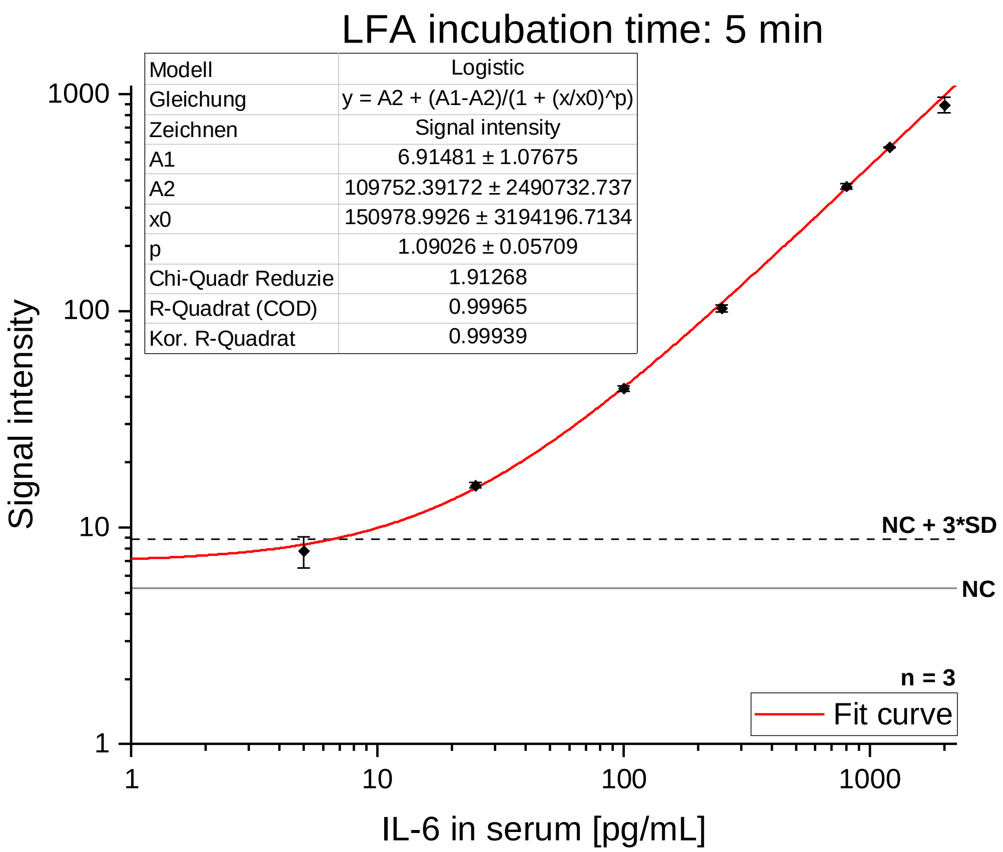

Figure S5. Measured points and fit curve of the centrifugal IL-6 assay after 5 min incubation time. The logistic fit curve model parameter are shown in the upper left corner.

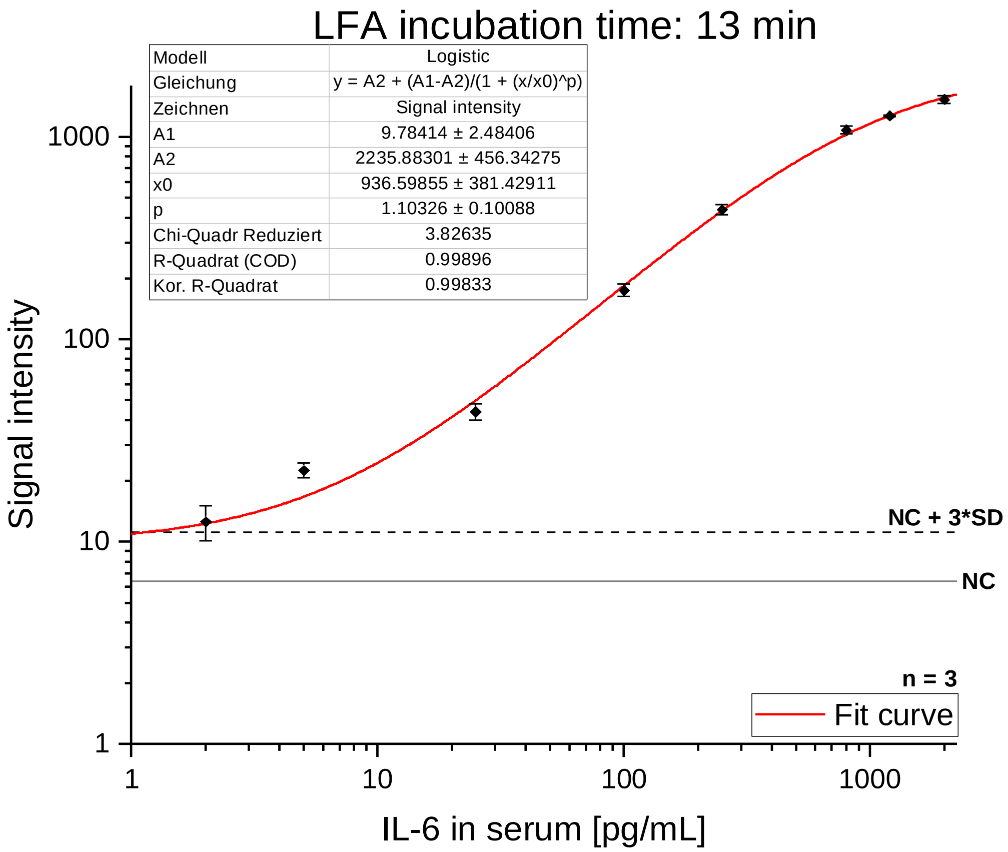

Figure S6. Measured points and fit curve of the centrifugal IL-6 assay after 13 min incubation time. The logistic fit curve model parameter are shown in the upper left corner.

Centrifugal gold nanoparticle lateral flow assay

For each test, 84 µL of spiked serum was mixed with 16 µL of an AuNP solution, which was provided by Milenia, resulting in a total sample volume of 100 µL. The 100 µL sample was then pipetted into a centrifugal microfluidic chip equipped with an integrated membrane. To ensure compatibility with the AuNP solution, the commercially available lateral flow strips from Milenia were cut to a length of 8 mm, ensuring that the test line was positioned at the center of the strip. These strips were then directly integrated into the microfluidic structure.

The rotational frequency was adjusted to achieve an incubation time of 20 minutes. After incubation, the test strips were automatically analyzed using a movable detector. The results are presented in Figure S7. The limit of detection (LoD) was determined to be 18.5 pg/mL, defining the assay's dynamic range between 18.5 pg/mL and approximately 4000 pg/mL.

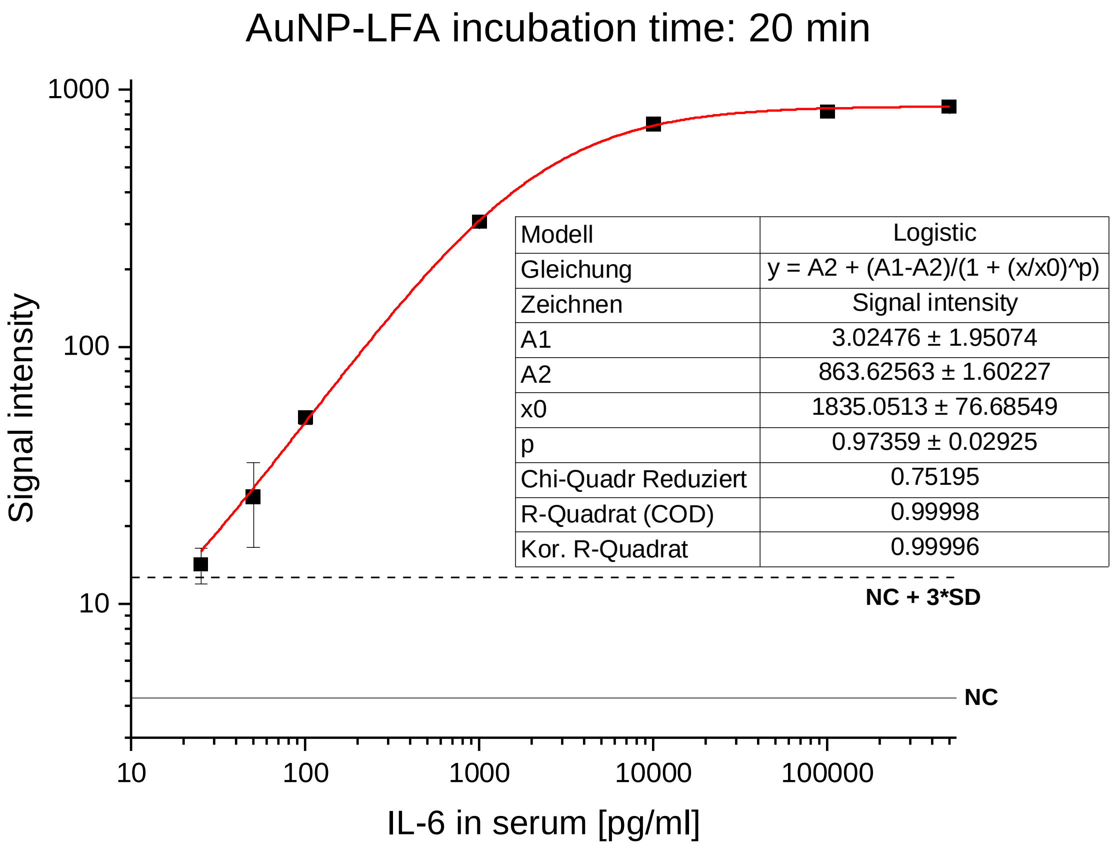

Figure S7. Measured points and fit curve of the centrifugal IL-6 AuNP assay after 20 min incubation time and a sample volume of 100 µl Serum. The logistic fit curve model parameter are shown in the lower right corner.
